# Nutritional status and psychosocial stimulation associated with cognitive development in preschool children: A cross-sectional study at Western Terai, Nepal

**DOI:** 10.1371/journal.pone.0280032

**Published:** 2023-03-13

**Authors:** Prakash Sharma, Chitra Bahadur Budhathoki, Ram Krishna Maharjan, Jitendra Kumar Singh

**Affiliations:** 1 Faculty of Education, Butwal Multiple Campus, Tribhuvan University, Butwal, Nepal; 2 Faculty of Education, Tribhuvan University, Kathmandu, Nepal; 3 Department of Community Medicine, Janaki Medical Collage, Tribhuvan University, Janakpur, Nepal; Agricultural University of Athens Department of Food Science and Human Nutrition: Geoponiko Panepistemio Athenon Tmema Epistemes Trophimon kai Diatrophes tou Anthropou, GREECE

## Abstract

Quality education at the age of foundation to produce dynamic manpower is a public concern in developing countries including Nepal. Preschool children do not get proper care and support from their parents due to insufficient knowledge of proper feeding habits, nutrition status and methods of psychosocial stimulation, which may affect their proper cognitive development. This study aimed to identify the factors that influence cognitive development in preschool children aged 3–5 years in Rupandehi district of western Terai, Nepal. In this school based cross-sectional survey, a total of 401 preschool children were selected using a multistage random sampling technique. The study was conducted from 4^th^ February to 12^th^ April, 2021 in Rupandehi district of Nepal. Data on the children’s socio-economic and demographic status, level of psychosocial stimulation, nutritional status, and stage of cognitive development were collected through scheduled interviews and direct observation. Stepwise regression analysis was performed to determine the predictors of cognitive development in preschool children. A *p*-value less than 0.05 considered as statistical significance. Of 401 participants, 44.1% had a normal nutritional status based on height for age Z-score (HAZ). Only 1.2% of primary caregivers provided their children with high levels of psychosocial stimulation, and 49.1% of children had a medium level of cognitive development. Furthermore, cognitive development in preschoolers is positively associated with nutritional status based on the height for age z score (β = 0.280; *p*<0.0001), psychological stimulation from caregivers (β = 0.184; *p*<0.0001), and advantageous castes/ethnicity (β = 0.190; *p*<0.0001), but negatively associated with the child’s age (β = - 0.145; *p* = 0.002) and family type (β = -0.157; *p* = 0.001). Nutritional status and psychosocial stimulation appear to be major factors affecting cognitive development of preschoolers. Nutritional promotion strategies, as well as techniques for optimal psychosocial stimulation behavior, may play an important role in enhancing preschoolers’ cognitive development.

## Introduction

As a child grows, it gains mental skills like perception, memory, the ability to solve problems, and the ability to learn new languages and dialects [1]. All of the processes by which sensory inputs are reduced, transformed, elaborated, stored, recovered and utilized are considered to as cognitive processes [2], which may be influenced by biological factors such as heredity and normality of sensory organs, living environment, social interaction, stimulating environment, adequate nutrients, poverty, motivating factors and adequate learning opportunities [3,4]. The first few years of a child’s life are vulnerable time for cognitive development which affects future educational and occupational possibilities, and it may also decide a person’s risk of physical health in terms of obesity, malnutrition and mental-health issues [5].

Nutrition plays a critical role in cell proliferation, DNA synthesis, hormone metabolism, and the production of neurotransmitters, all of which contribute to improved mental health [6]. On the other hand, children’s culture, psychosocial stimulation, and living environment influence the cognitive development process [7]. Psychosocial stimulation, sociocultural factors, and realistic deficiency all affect the development of skills like cognitive, motor, perceptual, and language abilities [8]. Early nutritional feeding and a healthy psychosocial stimulation environment can lead to major changes in physical and mental development that affect children’s cognition, mood, and behavior [2,9].

A community-based cohort study in the United States examined the long-term effect of a randomized intervention based on nutrition, supplementation and psychosocial stimulation for malnourished children and found improved school attendance and learning performance [10]. In an experimental study carried out in an upper-middle income country, Jamaica reported that the group that received only nutrition and the group that received only psychosocial stimulation showed less cognitive development than the group that received both nutrition and stimulation [11]. Another cohort study in the same country compared the cognitive scores of children born to stunted and non-stunted parents, and discovered that the cognitive scores of the children born to stunted parents were lower, indicating that the effects of stunting on cognitive development continued in the subsequent generation of children [12]. Preschoolers’ cognitive development is significantly influenced by their diet and home environment, according to a cross-sectional study done in a Costa Rican refugee camp [13]. In the context of developing nations, Santosh et al. recommended that psychosocial stimulation and adequate nutritional status should both be intervened for proper cognitive development of children [14].

However, there is limited information on the determinants of early child development in low-income countries, especially rural sub-Saharan Africa and South Asia, where children are at high risk of not reaching their developmental potential due to the presence of many risk factors for poor cognitive development [1,3,9]. Very few studies have examined the relationship between nutrition status, psychosocial stimulation, and cognitive development, and they suggested a study focusing on the interactive effects of nutrients and psychosocial stimulation on the cognitive development of preschool children [15]. Similarly, there is a little practical discussion in the study literature of the challenges faced by the growing child in the setting of Nepal in the process of cognitive development. The rationale behind the study is to examine whether preschoolers’ nutritional status and psychosocial stimulation enhance their cognitive development.

Therefore, this study was conducted to assess the effect of nutritional status and psychosocial stimulation on cognitive development among preschool children aged 3–5 years in Western Terai of Nepal.

## Methods

### Study design and setting

This study used a quantitative cross-sectional survey design with preschool children and their primary caregivers as respondents, in accordance with the post-positivist research paradigm of a single and objective reality.The study was conducted from 4^th^ February to 12^th^ April, 2021 in Rupandehi district of Nepal, that comprises one-metropolitan city, one municipality, and one rural municipality.

Located in Lumbini province, Rupandehi district has a total area of 1360 square kilometers and is divided into five electoral constituencies, ten provincial constituencies and sixteen local government units. It is also the birth place of Lord Buddha, lies in the western Terai of Nepal. The district had a population of 1,118,975 as of the most recent census, which was conducted in 2021. The population is diverse in terms of ethnicity, culture, and socio-economic background, and the district has a relatively high fertility rate, resulting in more children than in other places [16]. According to data obtained from the education division of the local government unit for the 2020 academic year, there were 14358 three- to five-year-olds children enrolled in 369 government funded early childhood development (ECD) centers [17].

### Participants and sampling approach

The study population consisted of preschool-aged children and their primary caregiver or mothers in the Rupandehi district. The study comprised three- to five-year-old children attended the ECD centre at the time of survey and whose mothers consented to their participation. Children were excluded if they had physical and mental problems that would affect the study. Raosoft’s sample size calculator was used to estimate a sample size based on the population size of 14358 three- to five-year children, an error margin of 5%, confidence level of 95%, and p (proportion of children who were deemed to be developing appropriately in the learning domain) as 82.4% [18], with a design effect of 1.7. A minimum sample of 375 was computed and 7% was added to account for non-responses, resulting in a sample size of 401. Multi-stage sampling was adopted to select the participants from Rupandehi district of Nepal. In the first stage, three local government units (one sub-metropolitan city, one municipality and one rural municipality) were selected at random in the district. These three local government units were chosen because they represent large urban and rural municipalities, resulting in a more representative sample. In the second stage, five ECD centres were chosen at random from a list provided by the education department in each local government unit. In the third stage, preschool children aged 3–5 years were selected using the population proportionate sampling (PPS) technique. Finally, a total of 401 preschool children (137 from sub-metropolitan city, 171 from municipality, and 93 from rural municipality) who were present at the EDC center at the time of the survey were enrolled in the study. Before enrolling children in the study, parental written consent was obtained. Participation rate between EDC center ranged from 97% to 100%.

### Data collection

Data were collected through scheduled interviews and direct observation. The data included the children’s socio-economic and demographic status, level of psychosocial stimulation, nutritional status, and stage of cognitive development. The socio-economic and demographic components included nine variables.The gender of the child, the number of children in the household, age, family type, caste/ethnicity, mother tongue, father’s education, mother’s education, and economic situation were all taken into consideration. The economic ranking tool was adopted from Nepal Demographic and Health Survey 2016 (NDHS) [19] to determine economic status. The wealth status was measured based on household assets (chair, bed, radio, television, cassette player, mobile, car, motorcycle, bicycle etc.). A housing index was made by rating condition of the roof, floor and wall of the house, fuel used to cook, types of latrine, and availability of water supply. The sum of the wealth scores was then utilized to determine economic status. The score of wealth quartile (- 0.54 to +3.48) was divided by 25% in each four categories: the poorest, poor, rich and the richest where <1.8 was poorest level of economic status. Likewise, from wealth quartile score >1.8 to <2.4 was categorized into poor, from >2.5 to <2.9 rich and >2.9+ were richest [17].

To determine the level of psychosocial stimulation, 37 questions based on cognitive stimulation, emotional stimulation, verbal responsiveness, avoidance of restriction and punishment, caregiver promotes child development, organization of physical and temporal environment, provision of appropriate play material, and opportunities for variety in daily stimulation were developed [3,20,21]. Out of thirty-seven questions, seven were eliminated in order to adjust Cronbach’s Alpha, and the final 28 questions were assessed with a binary response of either yes or no. One mark was assigned for a positive response and zero marks for a negative response. The total number and percentage of responses were calculated. Participants were divided into three levels of psychosocial stimulation: low (less than 52%), medium (between 53% and 82%), and high (more than or equal to 83%) based on literature [22]. The height and weight of preschool children were measured at their respective EDC centers. A SAMSO branded LCD digital weighing scale (L = 31cm,W = 29.4cm, and H = 2.65cm) with a capacity of 150kg x 100g and a minimum weighing capacity of 0.82kg was used to measure body weight. To avoid reading parallax and ensure accurate and precise measurement, a portable child height measuring wooden board with a smooth gliding measuring slide/wedge that could be locked or had a friction feature was used. Over its entire length, the measuring slide wobbled only 0.2 cm, making it possible to take numerous precise readings. Five technical experts with bachelor’s degrees in a health-related field who were trained for the study protocol, tools, and data collection methods collected the data.

The age, height, and weight of children were measured in accordance with WHO standards for child growth [23]. Using WHO Anthro plus software [24] version 1.0.4, the Z-scores of the index for height-for-age Z-score (HAZ), weight-for-age Z-score (WAZ), and body mass index Z-score (BAZ) were calculated. The HAZ, WAZ, and BAZ scores of the children were then divided into three levels of nutrition status according to WHO nutrition guidelines: normal (score between -1 SD and +1 SD), moderate malnutrition (-2 SD to -1 SD; or +1 SD +2 SD), and severe malnutrition (less than -2 SD or greater than +2 SD) [24]. The cognitive development of children was assessed using a tool developed by Hema Pandey in 1992, [25] which was later given to the National Psychological Corporation of India with copyright registered in 2005. The assessment tool has six dimensions: conceptual skill, information, comprehension, memory, visual perception, and object vocabulary [25]. All six aspects of cognitive development were represented by 20 items with a maximum possible score of 65, ranging from one to eight for each item score [26]. All of these aspects occur during the pre-operational stage, which was initiated by Jean Piaget in his developmental psychology [27]. Only a few of the 20 items were adjusted to contextualize them for the current situation.The raw score obtained by three, four, and five-year-old children was converted into a standard score using the cognitive development tools’ guidelines [26]. The total score was converted into percentages for low (<60%), medium (60–79%), and high (≥ 80%) categories [22], in compliance with Nepal’s national education grading system. In the Nepali educational system, there are eight grades, which are designated as A+, A, B+, B, C+, C, D, and E based on scores of 90% or higher, 80–89%, 70–79%, 60–69%, 50–59%, 40–49%, 25–39% and <25%, respectively [28,29]. Because of the wide range of grade levels, it was further classified into three categories: high (A and A+), medium (B and B+), and low (C+ and lower) [28].

### Reliability of tools

Tools for cognitive development, psychosocial stimulation and socio-economic and demographics were pre-tested among 10% of respondents who were not included in the study. Tools were pre-tested to determine their understanding, time spent on each question, and consistency among related variables and acceptability [30]. Data collection assistants were intensively trained on the objective of the study and techniques of data collection. Two-day training was provided to the enumerators with a mock session. They were facilitated about the potential biases during data collection and some techniques such as probing questions, logic patterns, and other appropriate skills were instructed them. The reliability of the cognitive development tool was 0.9 and 0.8 for psychosocial stimulation assessed by use of Cronbach’s alpha test [31]. The subjects’ privacy and self-confidence were respected during the interviews and cognitive development tests.

### Statistical analysis

The data were entered into an Excel sheet and then transferred to the version 26.0 of IBM SPSS for statistical analysis. Data were coded and categorized according to the needs of the objectives and nature of the variables. Categorical variables were presented as numbers (n) and percentages (%), whereas continuous variables were represented as means and standard deviations (SD). The T-test and one-way ANOVA with post hoc analysis were conducted. A p-value was considered significant if it was less than 0.05. The Kolmogorov-Smirnov test was used to determine the normality of the data, and nine outliers were eliminated from the dependent variable to make it normally distributed. Considered OLS assumptions were: (1) Normality of residuals utilizing the Histogram and PP plot; (2) Homogeneity; and (3) Multicollinearity. Cognitive development was predicted by twelve variables: HAZ, WAZ, BAZ, psychosocial stimulation, economic status, types of family, caste/ethnicity, language, mother’s education, father’s education, number of children, and age.

For the regression analysis, there were five possible models built from the stepwise selection method. The results of ANOVA depicted that the entire model has a significant overall fit to the given set of observations. The values of F were gradually decreased from the first model (37.98) to the fifth model (19.38) and also they were all highly significant (*p*<0.01) supporting them as the better models. The increased adjusted R square from first to last model as 08.8%, 13.5%, 15.9%, 17.5% and 19.4%, respectively, and the decreased standard error of the estimate from the model first to last as 16.34, 15.91, 15.69, 15.54 and 15.36 respectively were also indicated a good sign of fitting of better models. Finally, the decreased values of Akaike’s information criterion (AIC) as 3320.70, 3302.11, 3292.87, 3286.13 and 3277.34 and Bayesian information criterion (BIC) 3332.62, 3318.01, 3312.74, 3309.98 and 3305.16 from model 1 to 5 provided similar support for the good model.

### Ethical consideration

The study received ethical approval from ethical review board of the Nepal Health Research Council (NHRC: No. 2078-56/2021). Furthermore, permission for the study was taken from the office of the Dean, Faculty of Education, Tribhuvan University, as well as the individual selected ECD centers. Written informed consent was obtained from the primary caregivers of preschool-aged children.

## Results

Distribution of sample characteristics is shown in Table 1. Of the 401 preschoolers, more than half (52.6%) were from joint family, more than one third (34.9%) were from advantageous caste or ethnicity, nearly a quarter (23.9%) were from a non Dalit Terai caste, and more than half (62.3%) spoke Nepali as their first language. In regard to education, 14.7% of fathers and 23.7% of mothers of preschoolers were illiterate. Almost one fourth of the participants were either the poorest, poor, rich, or the richest, and the majority (71.6%) of parents had two or fewer children. Likewise, half of the children were male (50.6%) and 45.6% of them were five years old. The nutritional status of children measured by height for age z score (HAZ) showed that 44.1% of preschool children had normal nutritional status, while the remainder were stunted, with 36.2% moderate and 19.7% severe. The prevalence of underweight children was higher, with 36.4% in the moderate level and 18.5% in the severe level for WAZ, whereas 45.1% of the children were normal. The majority of the study’s children (57.1%) fell into the normal category according to their BMI-for-age (BAZ) classification, followed by the moderate category with 31.2% and the severe category with only 11.7%.

**Table 1:** Distribution of sample characteristics (n = 401).

Characteristics	Category	N	%
Types of family	Nuclear Joint	190 211	47.4 52.6
Caste/ethnicity*	Dalit Janajati Non-Dalit Terai caste Advantageous caste	53 112 96 140	13.2 27.9 23.9 34.9
Mother tongue/language	Nepali Bhojpuri Magar Other	250 104 16 31	62.3 25.9 4.0 7.7
Father’s education	Illiterate Basic Level Secondary and above	59 199 143	14.7 49.6 35.7
Mother’s education	Illiterate Basic Level Secondary and above	95 181 125	23.7 45.1 31.2
Economic status	Poorest Poorer Rich Richest	101 100 101 99	25.2 24.9 25.2 24.7
Number of children	Less than or equal to two More than two	287 114	71.6 28.4
Gender of children	Male Female	203 198	50.6 49.4
Age of children	Three years Four years Five years	39 179 183	9.7 44.6 45.6
Height for age (HAZ) score of children (Stunted)	Normal Moderate Severe	177 145 79	44.1 36.2 19.7
Weight for age (WAZ) score of children (Underweight)	Normal Moderate Severe	181 146 74	45.1 36.4 18.5
Body mass index (BMI) for age (BAZ) score of children (Wasting/thinness)	Normal Moderate Severe	229 125 47	57.1 31.2 11.7
Psychosocial stimulation by caregivers	Low Medium High	119 277 5	29.7 69.1 1.2
Cognitive development of children	Low Medium High	37 197 167	9.2 49.1 41.6

*Caste/ethnicity-Dalit is considered as extremely socially backward caste in Nepal with caste-based discrimination including untouchability. Advantageous caste are considered socially forward group with highest social status. Janajati and non-Dalit Tarai castes are considered in the rank of above Dalit and below the disadvantageous caste of social position [32].

Only 1.2% of primary caregivers provided high levels of psychosocial stimulation, compared to 69.1% who provided medium levels and 29.7% who provided low levels. The greatest proportion of children (49.1%) had a medium level of cognitive development, while 41.6% had low levels of cognitive development and the remaining (9.2%) high level of cognitive development.

### Cognitive development and determinant factors

Table 2 showed that the mean cognitive development score significantly differed by socio-economic and demographic, nutritional status, and psychological stimulation categories. There were significant differences in the mean cognitive development score by age category (*p* = 0.003), number of children (*p*<0.0001), family types (*p*<0.018), caste/ethnicity (p<0.0001), mother tongue/language (*p* = 0.015), father’s education (*p* = 0.011), mother’s education (*p* = 0.0001), economic status (*p* = 0.016), psychosocial stimulation (*p* = 0.002), HAZ classification (*p*<0.0001), WAZ classification (*p*<0.015), and BAZ classification (*p*<0.022).

**Table 2:** Analysis of cognitive development with determinant factors (n = 393).

Variables	Category	Number (%)	Mean	SD	*P*-value#
Age of children	Three years Four years Five years	35(8.9) 179(45.5) 179(45.5)	110.02 101.61 99.61	27.00 12.82 17.59	0.003***
Number of children	Less than or equal two More than two	282(71.8) 111(28.2)	102.38 100.29	15.53 15.97	0.0001***
Types of family	Nuclear Joint	186(47.3) 207(52.7)	103.75 100.02	14.69 16.32	0.018*
Caste/ethnicity	Dalit Janajati Non-Dalit Terai caste Advantageous caste	52(13.2) 110(28.0) 94(23.9) 137(34.9)	97.67 97.69 97.83 108.76	17.46 15.12 17.21 16.34	0.0001***
Mother tongue/language	Nepali Bhojpuri Magar Others	245(62.3) 101(25.7) 16(4.1) 31(7.9)	103.33 98.61 106.56 97.48	15.23 15.61 15.61 17.32	0.015*
Father’s education	Illiterate Basic Level Secondary and above	59(15.0) 195(49.6) 139(35.4)	99.77 99.56 105.02	13.62 17.39 17.67	0.011*
Mother’s education	Illiterate Basic Level Secondary and above	94(23.4) 180(45.8) 119(30.3)	97.73 100.25 106.45	16.63 16.99 16.78	0.0001***
Economic status	Poorest Poor Rich Richest	101(25.7) 98(24.9) 98(24.9) 96(24.4)	98.58 99.18 105.18 103.28	15.59 15.47 17.25 19.40	.016*
Psychosocial stimulation by caregivers	Low Medium High	116 (29.5) 272 (69.2) 5 (1.3)	97.67 103.40 109.20	15.67 15.37 15.53	0.002***
HAZ Classification	Normal Moderate Severe	172(43.8) 142(36.1) 79(20.1)	105.42 101.54 93.00	16.59 16.92 15.74	0.0001***
WAZ Classification	Normal Moderate Severe	176(44.8) 143(36.4) 74(18.8)	102.65 102.81 96.35	15.80 18.46 16.82	0.015*
BAZ Classification	Normal Moderate Severe	224(57.0) 122(31.0) 47 (12.0)	100.56 100.79 108.00	16.17 18.79 16.09	0.022*

Note: Significant at *p<0.05 and **p<0.01, ***p<0.0001, # p-value calculated for T-test and ONE WAY ANOVA.

### Stepwise regression analysis and determinant factors of cognitive development

The results of the stepwise regression analysis showed that psychological stimulation from caregivers, advantageous castes/ethnicity, and nutritional status based on the height for age z score (HAZ) (*p*<0.01) all had a positive and significant impact on cognitive development. The age of the child and living in a joint family, however, had a negative and significant effect (*p*< 0.01) [Table 3].

**Table 3:** Stepwise regression analysis and determinant factors of cognitive development.

Predictors	Model 1	Model 2	Model 3	Model 4	Model 5
HAZ	0.301** (0.723)	0.323** (0.707)	0.298** (0.706)	0.284** (0.702)	0.280** (0.695)
Advantageous castes/ethnicity		0.222** (1.656)	0.194** (1.657)	0.201** (1.643)	0.190** (1.628)
Psychosocial stimulation by care givers			0.165** (0.191)	0.185** (0.191)	0.184** (0.189)
Joint family				-0.137** (1.615)	-0.157** (1.611)
Age of child					-0.145** (1.211)
Constant	106.242** (1.128)	102.099** (1.416)	89.233** (3.991)	89.831** (3.957)	106.939** (6.725)
Adjusted R^2^	08.8%	13.5%	15.9%	17.5%	19.4%
Std. Error	16.34	15.91	15.69	15.54	15.36
F (*P*-value)	37.98 (*P*<0.01)	30.78 (*P*<0.01)	25.05 (*P*<0.01)	21.28 (*P*<0.01)	19.38 (*P*<0.01)
AIC^♀^	3320.70	3302.11	3292.87	3286.13	3277.34
BIC^♀^	3332.62	3318.01	3312.74	3309.98	3305.16

Dependent variable: Cognitive development.

Note: The figure in a parenthesis is standard error of an estimate.

♀Based on Maximum Likelihood estimation, Significance: ** at 1% and *at 5%.

According to this finding, nutritional status (HAZ) alone in model 1 can account for 8.8% of a child’s cognitive development (β = 0.301 and adjusted R^2^ = 0.088). When advantageous caste/ethnicity was included in model 2, the contribution rose by 13.5%. The effect of psychosocial stimulation was shared by 15.9% in model 3 and 17.5% when family type was included in model 4. Finally, the five factors, namely nutritional status (HAZ), caste/ethnicity, psychosocial stimulation, family type, and child age, explained 19.4% of a child’s cognitive development in model 5.

In the final multivariable regression model, a single unit change in height for age Z score (HAZ) resulted in a 0.280 unit change in cognitive development score (β = 0.280; *p*<0.0001). Similar to this, an increase of one unit in psychological stimulation was associated with an increase of 0.184 units in the cognitive development score (β = 0.184; *p*<0.0001). Additionally, changing caste/ethnicity categories resulted in a 0.190 unit change in cognitive development score (β = 0.190; *p*<0.0001). Furthermore, the cognitive development score dropped by 0.157 units (β = -0.157; *p* = 0.001) when the family type changed from nuclear to joint. Similar to this, for every unit increase in pre-schooler age, the cognitive development score dropped by 0.145 units (β = - 0.145; *p* = 0.002).

## Discussion

The study revealed that the gender ratio was almost equal, about fifty percent was in marginal economic condition and most of them were undernourished. Almost half of them were in medium level of cognitive development and most of them received a medium level of psychosocial stimulation. The age of the child, number of child, types of family, caste/ethnicity, mother tongue, parental education, economic status, psychosocial stimulation and nutrition status [HAZ, WAZ, BAZ] were the main predictors of cognitive development of preschoolers in unadjusted analysis. In multivariate analysis, this study showed that nutrition status [HAZ], advantageous caste and psychosocial stimulation had significant and positive relationship with cognitive development. However, a significant but negative relationship was found with joint family and age of children.

Nutritional status (HAZ) appeared to be the most important and positive contributing predictor. When comparing with nutrition status HAZ, cognitive development in preschool children has a considerable impact since children who were undernourished have lower levels of cognitive growth. Cognitive development had a substantial impact on nutritional status as children with intermediate and severe nutrition status were reported to have low levels of cognitive development. It was associated with development and performance on social perception tests and visual-spatial abilities at 5 years of age [33] while studying the effect of vitamin B-12 in cognitive functioning among children from the Bhaktapur district of Nepal. Similarly, undernourishment and non-verbal IQ were found to be substantially associated with South-East Asian children aged 6 to 12 years [34]. In Bangladesh, a randomized controlled experiment comparing children aged 6 to 24 months with and without nutritional supplement revealed a significant cognitive development benefit of nutrition [35]. The most important takeaway from this study was that preschoolers with moderate or severe malnutrition showed lower cognitive development. It indicated that a high degree of cognitive development in preschool children was linked to a better nutritional status as undernourishment creates impaired growth and dysfunction of neurocognition [36].

Caste/ethnicity affected cognitive development of preschoolers. Advantageous caste children had higher cognitive development than disadvantageous caste. In India, children from high castes had higher educational outcomes than those from lower castes such as Dalits, Adivasi, Muslims, and children from privileged castes. The children from the advantageous castes had enjoyed over their friends from the disadvantageous castes [37]. It indicated that parents of disadvantageous castes (Dalit, non-Dalit Terai caste, and Janajati) seemed to have less attention for the cognitive development of their preschool children. This could be because most of them were illiterate and busy for their livelihood, and most ECD teachers were from advantageous castes who might not understand the feeling and be insensitive to those children [37].

Preschool children’s cognitive development was significantly influenced by the psychosocial stimulation they received from their caregivers. It appeared that the cognitive development of children who received high and medium levels of psychosocial stimulation was superior to that of those who have received low levels. A randomized trial in Bangladesh comparing children aged 6–24 months with and without stimulation demonstrated a significant (p = 0.037) effect of stimulation on cognitive development [38]. Cognitive development was strongly influenced by the types of families a child grows up in. Preschool children’s cognitive development was reported to be better in nuclear families than joint families. The role of the nuclear family had a smaller and favorable impact compared to HAZ, the advantageous caste, and psychological stimulation. Research in Cuba indicated that parents from a nuclear family provided more attention to their children’s education and all-round development than parents from other families, which had an impact on their children’s performance [39]. A higher level of parent-child interaction, maternal care, and children’s autonomy was also found to be associated with nuclear families [40]. It may be the reason that it was difficult to provide appropriate nutrition and good practice of psychosocial stimulation such as sufficient play materials and mother-child interaction in joint family [41] as insufficient nutrition and poor psychosocial stimulation were directly associated with cognitive outcomes [41]. This means that children from a nuclear family had a better chance of developing their cognitive potential.

The cognitive development of children was influenced by their age. While other factors influence preschoolers’ cognitive development, age alone has the least and significant negative impact on their progress. It was found that as preschoolers grew older, their cognitive development slowed down, which went against the theory behind this study which might be the reason they had to deal with the responsibility of caring for young babies and doing household work [25]. A similar conclusion was obtained in a longitudinal follow-up research with 79 children from birth to 3.6 years old, which discovered that children who were exposed to low and high biological risk and had less home stimulation had lower cognitive development [42].

### Strength and limitation of the study

The strength of this study was its large sample size of 401 preschoolers and primary caregivers. Dr. Hema Pandey’s cognitive development tool was shown to be successful following contextualization and pre-testing in this population. Dietary status, psychosocial stimulation, and cognitive development were categorized based on WHO nutrition guidelines and literature review. This study filled a knowledge gap on the cognitive development of children in Nepal and South Asia. The findings of this study may guide academics and policymakers in developing and implementing formal and non-formal curriculum and recommendations to promote the cognitive development of preschoolers. However, there were some limitations of this study that need to be considered. Since the study was cross-sectional the whole data were obtained only on the day of survey. The study was conducted among children who studied in government ECD centers, so it may not be generalized to other preschoolers. This study did not assess the IQ of mothers/primary caregivers, which may influence child care and stimulation. Further, some of the determinants of child development such as genetic factors, family conflict and violence were not considered. The efforts of ECD centers and ECD teachers regarding nutrition education and practice as well as psychosocial stimulation behavior were not addressed in this study, which could have influenced its findings. Additionaly, the psychosocial stimulation measurement tool was created for local use and the score has not yet been validated; however tertiles were used for the analysis to assess trend [43].

## Conclusions

This study showed that some socio-economic and demographic factors, including the children’s age, caste/ethnicity, and family type, were significantly associated with preschoolers’ cognitive development. Furthermore, the cognitive development of preschoolers was likely to be significantly influenced by nutritional status and psychosocial stimulation. In the current context of Western Terai, Nepal, nutritional promotion strategies along with techniques of optimal psychosocial stimulation behavior for preschoolers mothers from disadvantageous groups (Dalit, non-Dalit Terai caste, and Janajati caste/ethnicity), as well as those living in joint families, may be a significant contributor in enhancing the cognitive development of preschoolers. It is essential to conduct additional research with a strong design and a large sample size to establish cause and effect relationship between nutritional status with psychosocial stimulation and cognitive development of preschoolers.

## Supporting information

S1 Checklist STROBE checklist cross-sectional. (DOCX)

S1 File Additional file- multiple linear regression results for cogninitive development. (DOCX)
