# Supplementary material for: Nutritional status and psychosocial stimulation associated with cognitive development in preschool children: A cross-sectional study at Western Terai, Nepal
Source: PLoS One. 2023 Mar 13;18(3):e0280032. doi: 10.1371/journal.pone.0280032 (PMC10010513; doi:10.1371/journal.pone.0280032)
Supplement: S1 File — (DOCX) [file pone.0280032.s002.docx]

**Additional file:**

**Table S2 (A). Analysis of cognitive development with determinant factors (n=393).**

| Variables | Category | Number | Mean | SD | Statistical method used | *P*-value |
| --- | --- | --- | --- | --- | --- | --- |
| Age of children | Three years  Four years  Five years | 35  179  179 | $110.02$  $101.61$  $99.61$ | 27.00  12.82  17.59 | One Way ANOVA | 0.003*** |
| Number of children | Less than or equal two  More than two | 282  111 | 102.38  100.29 | 15.53  15.97 | Independent sample T-test | 0.0001*** |
| Types of family | Nuclear  Joint | 186  207 | 103.75  100.02 | 14.69  16.32 | Independent T- sample test | 0.018* |
| Caste/ethnicity | Dalit  Janajati  Non-Dalit Terai caste  Advantageous caste | 52  110  94  137 | $97.67$  $97.69$  $97.83$  $108.76$ | 17.46  15.12  17.21  16.34 | One Way ANOVA | 0.0001*** |
| Mother tongue/language | Nepali  Bhojpuri  Magar  Others | 245  101  16  31 | 103.33  98.61  106.56  97.48 | 15.23  15.61  15.61  17.32 | One Way ANOVA | 0.015* |
| Father’s education | Illiterate  Basic Level  Secondary and above | 59  195  139 | 99.77  99.56  105.02 | 13.62  17.39  17.67 | One Way ANOVA | 0.011* |
| Mother’s education | Illiterate  Basic Level  Secondary and above | 94  180  119 | 97.73  100.25  106.45 | 16.63  16.99  16.78 | One Way ANOVA | 0.0001*** |
| Economic status | Poorest  Poor  Rich  Richest | 101  98  98  96 | 98.58  99.18  105.18  103.28 | 15.59  15.47  17.25  19.40 | One Way ANOVA | .016* |
| Psychosocial stimulation by caregivers | Low  Medium  High | 116  272  5 | 97.67  103.40  109.20 | 15.67  15.37  15.53 | One Way ANOVA | 0.002*** |
| HAZ Classification | Normal  Moderate  Severe | 172  142  79 | 105.42  101.54  93.00 | 16.59  16.92  15.74 | One Way ANOVA | 0.0001*** |
| WAZ Classification | Normal  Moderate  Severe | 176  143  74 | 102.65  102.81  96.35 | 15.80  18.46  16.82 | One Way ANOVA | 0.015* |
| BAZ Classification | Normal  Moderate  Severe | 224  122  47 | 100.56  100.79  108.00 | 16.17  18.79  16.09 | One Way ANOVA | 0.022* |

**Table S2 (B) - Multiple linear regression results for cognitive development**

| **Variables Entered/Removed^a^** | | | |
| --- | --- | --- | --- |
| Model | Variables Entered | Variables Removed | Method |
| 1 | HAZ | . | Stepwise (Criteria: Probability-of-F-to-enter <= .050, Probability-of-F-to-remove >= .100). |
| 2 | Advantageous caste | . | Stepwise (Criteria: Probability-of-F-to-enter <= .050, Probability-of-F-to-remove >= .100). |
| 3 | Psychosocial stimulation by caregivers | . | Stepwise (Criteria: Probability-of-F-to-enter <= .050, Probability-of-F-to-remove >= .100). |
| 4 | Joint family | . | Stepwise (Criteria: Probability-of-F-to-enter <= .050, Probability-of-F-to-remove >= .100). |
| 5 | Age of child | . | Stepwise (Criteria: Probability-of-F-to-enter <= .050, Probability-of-F-to-remove >= .100). |
| a. Dependent Variable: Cognitive development | | | |

| **Model Summary** | | | | |
| --- | --- | --- | --- | --- |
| Model | R | R Square | Adjusted R Square | Std. Error of the Estimate |
| 1 | .301^a^ | .090 | .088 | 16.34199 |
| 2 | .373^b^ | .139 | .135 | 15.91938 |
| 3 | .406^c^ | .165 | .159 | 15.69752 |
| 4 | .428^d^ | .183 | .175 | 15.54513 |
| 5 | .452^e^ | .204 | .194 | 15.36807 |
| a. Predictors: (Constant), HAZ | | | | |
| b. Predictors: (Constant), HAZ, Advantageous caste | | | | |
| c. Predictors: (Constant), HAZ, Advantageous caste, Psychosocial stimulation by caregivers | | | | |
| d. Predictors: (Constant), HAZ, Advantageous caste, Psychosocial stimulation by caregivers, Joint family | | | | |
| e. Predictors: (Constant), HAZ, Advantageous caste, Psychosocial stimulation by caregivers, Joint family, Age of child | | | | |

| **ANOVA^a^** | | | | | | |
| --- | --- | --- | --- | --- | --- | --- |
| Model | | Sum of Squares | df | Mean Square | F | Sig. |
| 1 | Regression | 10143.545 | 1 | 10143.545 | 37.982 | .000^b^ |
|  | Residual | 102017.195 | 382 | 267.061 |  |  |
|  | Total | 112160.740 | 383 |  |  |  |
| 2 | Regression | 15605.169 | 2 | 7802.585 | 30.788 | .000^c^ |
|  | Residual | 96555.570 | 381 | 253.427 |  |  |
|  | Total | 112160.740 | 383 |  |  |  |
| 3 | Regression | 18524.074 | 3 | 6174.691 | 25.058 | .000^d^ |
|  | Residual | 93636.666 | 380 | 246.412 |  |  |
|  | Total | 112160.740 | 383 |  |  |  |
| 4 | Regression | 20575.017 | 4 | 5143.754 | 21.286 | .000^e^ |
|  | Residual | 91585.722 | 379 | 241.651 |  |  |
|  | Total | 112160.740 | 383 |  |  |  |
| 5 | Regression | 22885.640 | 5 | 4577.128 | 19.380 | .000^f^ |
|  | Residual | 89275.099 | 378 | 236.178 |  |  |
|  | Total | 112160.740 | 383 |  |  |  |
| a. Dependent Variable: Cognitive development | | | | | | |
| b. Predictors: (Constant), HAZ | | | | | | |
| c. Predictors: (Constant), HAZ, Advantageous caste | | | | | | |
| d. Predictors: (Constant), HAZ, Advantageous caste, Psychosocial stimulation by caregivers | | | | | | |
| e. Predictors: (Constant), HAZ, Advantageous caste, Psychosocial stimulation by caregivers, Joint family | | | | | | |
| f. Predictors: (Constant), HAZ, Advantageous caste, Psychosocial stimulation by caregivers, Joint family, Age of child | | | | | | |

| **Coefficients^a^** | | | | | | | | |
| --- | --- | --- | --- | --- | --- | --- | --- | --- |
| Model | | Unstandardized Coefficients | | Standardized Coefficients | t | Sig. | Collinearity Statistics | |
|  |  | B | Std. Error | Beta |  |  | Tolerance | VIF |
| 1 | (Constant) | 106.242 | 1.128 |  | 94.154 | .000 |  |  |
|  | HAZ | 4.454 | .723 | .301 | 6.163 | .000 | 1.000 | 1.000 |
| 2 | (Constant) | 102.099 | 1.416 |  | 72.115 | .000 |  |  |
|  | HAZ | 4.779 | .707 | .323 | 6.755 | .000 | .990 | 1.010 |
|  | Advantageous caste | 7.687 | 1.656 | .222 | 4.642 | .000 | .990 | 1.010 |
| 3 | (Constant) | 89.233 | 3.991 |  | 22.361 | .000 |  |  |
|  | HAZ | 4.413 | .706 | .298 | 6.253 | .000 | .968 | 1.033 |
|  | Advantageous caste | 6.722 | 1.657 | .194 | 4.057 | .000 | .962 | 1.040 |
|  | Psychosocial stimulation by caregivers | .658 | .191 | .165 | 3.442 | .001 | .954 | 1.048 |
| 4 | (Constant) | 89.831 | 3.957 |  | 22.701 | .000 |  |  |
|  | HAZ | 4.213 | .702 | .284 | 6.000 | .000 | .958 | 1.043 |
|  | Advantageous caste | 6.954 | 1.643 | .201 | 4.234 | .000 | .960 | 1.042 |
|  | Psychosocial stimulation by caregivers | .736 | .191 | .185 | 3.849 | .000 | .936 | 1.069 |
|  | Joint family | -4.705 | 1.615 | -.137 | -2.913 | .004 | .968 | 1.033 |
| 5 | (Constant) | 106.939 | 6.725 |  | 15.903 | .000 |  |  |
|  | HAZ | 4.140 | .695 | .280 | 5.961 | .000 | .957 | 1.044 |
|  | Advantageous caste | 6.580 | 1.628 | .190 | 4.041 | .000 | .954 | 1.048 |
|  | Psychosocial stimulation by caregivers | .732 | .189 | .184 | 3.876 | .000 | .936 | 1.069 |
|  | Joint family | -5.380 | 1.611 | -.157 | -3.339 | .001 | .951 | 1.051 |
|  | Age of child | -3.789 | 1.211 | -.145 | -3.128 | .002 | .974 | 1.026 |
| a. Dependent Variable: Cognitive development | | | | | | | | |

| **Excluded Variables^a^** | | | | | | | | |
| --- | --- | --- | --- | --- | --- | --- | --- | --- |
| Model | | Beta In | t | Sig. | Partial Correlation | Collinearity Statistics | | |
|  |  |  |  |  |  | Tolerance | VIF | Minimum Tolerance |
| 1 | Psychosocial stimulation by caregivers | .198^b^ | 4.106 | .000 | .206 | .982 | 1.018 | .982 |
|  | WAZ | -.111^b^ | -1.604 | .110 | -.082 | .496 | 2.017 | .496 |
|  | BAZ | -.087^b^ | -1.789 | .074 | -.091 | 1.000 | 1.000 | 1.000 |
|  | Economic ranking | .056^b^ | 1.118 | .264 | .057 | .948 | 1.055 | .948 |
|  | Joint family | -.095^b^ | -1.955 | .051 | -.100 | .993 | 1.007 | .993 |
|  | Language/mother tongue | .123^b^ | 2.514 | .012 | .128 | .974 | 1.027 | .974 |
|  | Mothers’ Education | .108^b^ | 2.215 | .027 | .113 | .996 | 1.004 | .996 |
|  | Cast of respondent | .222^b^ | 4.642 | .000 | .231 | .990 | 1.010 | .990 |
|  | Fathers’ education | .045^b^ | .932 | .352 | .048 | 1.000 | 1.000 | 1.000 |
|  | Number of child | -.024^b^ | -.489 | .625 | -.025 | .991 | 1.009 | .991 |
|  | Age of child | -.146^b^ | -3.028 | .003 | -.153 | 1.000 | 1.000 | 1.000 |
| 2 | Psychosocial stimulation by caregivers | .165^c^ | 3.442 | .001 | .174 | .954 | 1.048 | .954 |
|  | WAZ | -.074^c^ | -1.088 | .277 | -.056 | .488 | 2.048 | .488 |
|  | BAZ | -.062^c^ | -1.290 | .198 | -.066 | .986 | 1.014 | .976 |
|  | Economic ranking | .073^c^ | 1.489 | .137 | .076 | .943 | 1.061 | .941 |
|  | Joint family | -.112^c^ | -2.357 | .019 | -.120 | .988 | 1.012 | .984 |
|  | Language/mother tongue | .140^c^ | 2.938 | .004 | .149 | .969 | 1.032 | .967 |
|  | Mothers’ Education | .112^c^ | 2.370 | .018 | .121 | .995 | 1.005 | .986 |
|  | Fathers’ education | .043^c^ | .905 | .366 | .046 | 1.000 | 1.000 | .990 |
|  | Number of child | -.065^c^ | -1.333 | .183 | -.068 | .961 | 1.041 | .960 |
|  | Age of child | -.128^c^ | -2.715 | .007 | -.138 | .993 | 1.007 | .983 |
| 3 | WAZ | -.079^d^ | -1.181 | .238 | -.061 | .488 | 2.049 | .488 |
|  | BAZ | -.065^d^ | -1.381 | .168 | -.071 | .985 | 1.015 | .948 |
|  | Economic ranking | -.007^d^ | -.127 | .899 | -.007 | .734 | 1.363 | .734 |
|  | Joint family | -.137^d^ | -2.913 | .004 | -.148 | .968 | 1.033 | .936 |
|  | Language/mother tongue | .102^d^ | 2.062 | .040 | .105 | .890 | 1.124 | .877 |
|  | Mothers’ Education | .067^d^ | 1.341 | .181 | .069 | .889 | 1.125 | .853 |
|  | Fathers’ education | .023^d^ | .480 | .631 | .025 | .983 | 1.017 | .939 |
|  | Number of child | -.027^d^ | -.553 | .580 | -.028 | .907 | 1.102 | .901 |
|  | Age of child | -.125^d^ | -2.669 | .008 | -.136 | .992 | 1.008 | .954 |
| 4 | WAZ | -.081^e^ | -1.223 | .222 | -.063 | .488 | 2.049 | .488 |
|  | BAZ | -.066^e^ | -1.420 | .156 | -.073 | .985 | 1.015 | .935 |
|  | Economic ranking | -.015^e^ | -.278 | .781 | -.014 | .732 | 1.366 | .727 |
|  | Language/mother tongue | .074^e^ | 1.470 | .142 | .075 | .847 | 1.181 | .842 |
|  | Mothers’ Education | .044^e^ | .890 | .374 | .046 | .866 | 1.155 | .823 |
|  | Fathers’ education | .019^e^ | .407 | .684 | .021 | .982 | 1.018 | .920 |
|  | Number of child | -.019^e^ | -.389 | .698 | -.020 | .904 | 1.106 | .881 |
|  | Age of child | -.145^e^ | -3.128 | .002 | -.159 | .974 | 1.026 | .936 |
| 5 | WAZ | -.114^f^ | -1.723 | .086 | -.088 | .477 | 2.095 | .477 |
|  | BAZ | -.089^f^ | -1.904 | .058 | -.098 | .966 | 1.036 | .935 |
|  | Economic ranking | -.024^f^ | -.453 | .651 | -.023 | .730 | 1.371 | .726 |
|  | Language/mother tongue | .072^f^ | 1.450 | .148 | .074 | .846 | 1.181 | .842 |
|  | Mothers’ Education | .035^f^ | .715 | .475 | .037 | .863 | 1.159 | .823 |
|  | Fathers’ education | .028^f^ | .596 | .551 | .031 | .979 | 1.021 | .919 |
|  | Number of child | -.011^f^ | -.231 | .818 | -.012 | .902 | 1.109 | .881 |
| a. Dependent Variable: Cognitive total score | | | | | | | | |
| b. Predictors in the Model: (Constant), HAZ | | | | | | | | |
| c. Predictors in the Model: (Constant), HAZ, Advantageous caste | | | | | | | | |
| d. Predictors in the Model: (Constant), HAZ, Advantageous caste, Psychosocial stimulation by caregivers | | | | | | | | |
| e. Predictors in the Model: (Constant), HAZ, Advantageous caste, Psychosocial stimulation by caregivers, Joint family | | | | | | | | |
| f. Predictors in the Model: (Constant), HAZ, Advantageous caste, Psychosocial stimulation by caregivers, Joint family, Age of child | | | | | | | | |

| **Collinearity Diagnostics^a^** | | | | | | | | | |
| --- | --- | --- | --- | --- | --- | --- | --- | --- | --- |
| Model | Dimension | Eigenvalue | Condition Index | Variance Proportions | | | | | |
|  |  |  |  | (Constant) | HAZ | Advantageous caste | Psychosocial stimulation by caregivers | Joint family | Age of child |
| 1 | 1 | 1.674 | 1.000 | .16 | .16 |  |  |  |  |
|  | 2 | .326 | 2.265 | .84 | .84 |  |  |  |  |
| 2 | 1 | 2.336 | 1.000 | .05 | .07 | .06 |  |  |  |
|  | 2 | .449 | 2.281 | .02 | .76 | .31 |  |  |  |
|  | 3 | .215 | 3.298 | .93 | .17 | .63 |  |  |  |
| 3 | 1 | 3.208 | 1.000 | .00 | .03 | .03 | .00 |  |  |
|  | 2 | .474 | 2.602 | .00 | .87 | .12 | .00 |  |  |
|  | 3 | .296 | 3.291 | .02 | .04 | .85 | .03 |  |  |
|  | 4 | .021 | 12.259 | .97 | .06 | .00 | .97 |  |  |
| 4 | 1 | 3.810 | 1.000 | .00 | .02 | .02 | .00 | .02 |  |
|  | 2 | .488 | 2.794 | .00 | .86 | .02 | .00 | .12 |  |
|  | 3 | .411 | 3.045 | .00 | .03 | .40 | .00 | .61 |  |
|  | 4 | .270 | 3.760 | .03 | .03 | .56 | .04 | .25 |  |
|  | 5 | .021 | 13.372 | .96 | .06 | .00 | .96 | .00 |  |
| 5 | 1 | 4.722 | 1.000 | .00 | .01 | .01 | .00 | .01 | .00 |
|  | 2 | .492 | 3.098 | .00 | .91 | .01 | .00 | .07 | .00 |
|  | 3 | .416 | 3.367 | .00 | .02 | .25 | .00 | .73 | .00 |
|  | 4 | .327 | 3.799 | .00 | .01 | .70 | .01 | .14 | .01 |
|  | 5 | .034 | 11.824 | .02 | .04 | .02 | .79 | .03 | .20 |
|  | 6 | .009 | 23.129 | .98 | .01 | .01 | .19 | .02 | .79 |
| a. Dependent Variable: Cognitive development | | | | | | | | | |
